# Framework for Improving Patient Safety: Reference Model for FHIR-Enabled, Patient-Centric Home Medication List Management and Medication Reconciliation

**DOI:** 10.1055/a-2599-4135

**Published:** 2025-09-19

**Authors:** Noah D. Bastola, James E. Tcheng, David M. Schlossman, John R. Windle

**Affiliations:** 1 Department of Medicine, University of Nebraska Medical Center, Omaha, Nebraska, United States; 2 Department of Medicine, Duke University, Durham, North Carolina, United States; 3 Department of Health Services Administration, University of Alabama at Birmingham, United States

**Keywords:** medication reconciliation, health information interoperability, patient-generated health data, health information exchange, electronic health records

## Abstract

**Background:**

The Health Level 7 (HL7) Electronic Health Record Workgroup identified home medication list reconciliation as a prime opportunity to improve patient safety and reduce clinician burden. We developed a platform-neutral, Fast Healthcare Interoperability Resources (FHIR)-enabled reference model and demonstration wireframe to articulate the concepts of an interoperable, patient-centric home medication list management ecosystem.

**Methods:**

Four principal artifacts describe the reference model: (1) a conceptual (high-level) model, (2) a data architecture (detailed) model including representations of the interactions among actors, workflows, data, and functionality, (3) a functionality (style) guide describing expected system behaviors, and (4) a high-fidelity, end-to-end wireframe. The wireframe was constructed using JavaScript, Bootstrap Studio, and FHIR to maximize code modularity, device compatibility, and interoperability.

**Results:**

The conceptual and architecture models capture the complex interplay of actors and data occurring among healthcare providers, information systems, and patients, positioning the patient at the center of home medication list management. The style guide reflects functionality requirements. The wireframe demonstrates the use of FHIR for data interoperability while representing patient and clinician interactions that reduce burden. The wireframe accesses standardized data elements via FHIR calls to an EHR sandbox and integrates RxNorm content to improve usability and associated medication metadata. Finally, the wireframe generates a FHIR patient-reconciled medication list data package and printable lists that can be shared with the clinician to facilitate outpatient medication reconciliation.

**Conclusion:**

This proof-of-concept highlights the potential of FHIR to facilitate patient-facing medication list management and provides a reference framework for developers.

## Background and Significance

It is estimated that upwards of 50,000 people die each year in the United States due to preventable medication errors and an additional 1.3 million people per year are injured. 1 2 3 Medication errors occur for a wide variety of reasons, including incorrect or inappropriate dosing, poor anticipation of drug interactions or side effects by the prescriber (e.g., prescribing Beers List medications to the elderly), incorrect administration by the patient (e.g., neglecting or incorrectly taking a medication), and failure to document new, changed, omitted, or discontinued medications by the patient or prescriber. 2 4 5 6 Inaccuracies on medication lists are common, with studies reporting that upwards of 80% of medication lists contain errors. 7 8

Medication reconciliation is a process that requires both patient and provider to agree upon a medication list that accurately represents what the patient is actually taking. Breakdowns in this agreement can jeopardize patient safety and worsen disease management outcomes. 9 10 11 12 However, the process of medication reconciliation is highly burdensome and reduces the amount of time available for direct patient care. 13 14 As electronic healthcare records (EHRs) have become ubiquitous, digital approaches to coordinating and communicating accurate medication list information from patient to clinician are now possible.

Health Level 7 (HL7) is an international standards development organization responsible for developing and governing healthcare data interoperability standards, including Fast Healthcare Interoperability Resources (FHIR). FHIR is an open-source healthcare data standard maintained and developed through consensus decision-making by formally elected HL7 members consisting of key stakeholders in the healthcare community, including EHR developers, hospital leaders, insurers, and medical professionals. 15 16 The 21st Century Cures Act, enacted in December 2016, established FHIR as the standard for healthcare data interoperability and prioritized addressing clinician burden related to the use of EHRs. 17 In response, the HL7 Electronic Health Record Workgroup convened a task force, the Reducing Clinician Burden (EHR-RCB) workgroup, to identify potential approaches to reducing clinician burden within the context of healthcare data interoperability standards. A specific health technology gap identified by the workgroup was the need for a patient-centric, patient-facing, home medication list management ecosystem that could convey the patient medication list to the clinician and facilitate EHR reconciliation in the context of an outpatient office visit. 18 19 A low fidelity wireframe was developed by the EHR-RCB workgroup that expressed a limited subset of these functionalities. 20

Our current project extends that preliminary work completed by the HL7 EHR-RCB workgroup. We created a model to address two primary objectives: (1) improvement of the accuracy of the medication list from the patient's perspective (i.e., medications “as taken”) and (2) reduction of clinician burden associated with the medication reconciliation process. This model was then evaluated through the creation of a high-fidelity wireframe to demonstrate proof-of-concept feasibility, identify gaps in healthcare interoperability infrastructure, and inform the development of extensions to the current HL7 Electronic Health Record Functional Model and Personal Health Record Functional Model to incorporate patient-centric medication list management. 21 22

## Materials and Methods

The HL7 EHR-RCB workgroup previously created a series of artifacts specific to the home medication reconciliation use case, including a fictional narrative that detailed the flow of medication information between actors (patient, medical assistant, and clinician) typically involved in an outpatient primary care visit. 18 The narrative is written in a first person (patient) perspective and highlights common points of patient and clinician burden, failures in the current medication reconciliation process, and lack of interoperability with current systems used in healthcare. Starting with this narrative description, we identified four principal artifacts needed to fully model a patient-centric home medication list management system for outpatient clinical medication reconciliation: (1) a conceptual (high-level) model, (2) a data architecture (detailed) model, including representations of the interactions among actors, workflows, data, and functionality, (3) a functionality (style) guide describing expected systems behaviors, and (4) a high-fidelity, end-to-end wireframe that applies design specifications detailed in the generated artifacts.

In modeling the workflows, dataflows, information systems, and actors we recognized two key features necessary for the modeled medication reconciliation process. These features included providing patients with a list of medications identified as “active” in the clinician-facing EHR and other pharmacy information management systems, and the generation of a FHIR data package representing a patient-generated medication reconciliation event reflecting the “as taken” perspective. Possible patient-generated reconciliation data include confirmation of medications as prescribed, addition of new medications, patient annotation of existing medications, and removal of medications from their personal medication list. 23 Because of an anticipated lack of a mechanism for ingestion of a medication reconciliation data package via FHIR, we also specified the ability to print a summary of the patient-generated documentation to physically share with the clinician.

### Modeling Tools

The conceptual (high-level) and data architecture (detailed) representations were authored using the Lucid Chart application suite (Lucid Software, South Jordan, UT). The intent of the conceptual model was to provide contextual understanding of how a developed application would fit into patient and clinician workflows, while the intent of the data architecture model was to provide a blueprint for intended data flow through the developed application, through integrations with FHIR and other external application programming interfaces (APIs), and model anticipated interactions with actors and actor workflows. The data architecture model includes key data elements required to enable the envisioned functionality.

We extended the low-fidelity mockup previously designed by the HL7 EHR-RCB workgroup by modeling functionality and usability through creation of a high-fidelity wireframe. To develop this wireframe, we selected Bootstrap Studio (Bootstrap Studio, Varna, Bulgaria), a “drag and drop” development environment for quickly building web application interfaces. It concurrently created HTML and CSS code for display on both mobile and desktop platforms (i.e., responsive design) and could directly transfer the HTML and CSS into production development environments. Data fields for medication data elements were selected based on the HL7/NCPDP Standardized Medication Profile, release 1 and the United States Core for Data Interoperability (USCDI) Version 4 standards for medications (e.g., medication name, dose, and units). 24 25

### Wireframe Development

Using Bootstrap Studio, we produced an initial series of static HTML pages and respective CSS files to create the skeleton of the user interface. The next step was to develop the button logic and API calls necessary to populate the solution with simulated data from an EHR. We chose the Epic (Epic Systems, Verona, WI) FHIR Sandbox as our data source for the prototyping environment. 26 The JavaScript framework React.js (Meta, Menlo Park, CA) was used to develop the user interface given the component-based approach of React and the ability to directly import the HTML and CSS code created in Bootstrap Studio, the large number of available libraries, and the large body of existing technical support. The initial setup of the React environment was accomplished through the “create-react-app” package ( https://github.com/facebook/create-react-app ). To maintain a consistent application state across all screens, we opted to use the open-source Redux state management library EasyPeasy ( https://github.com/ctrlplusb/easy-peasy ) to create a single local data source for called data to be stored and for all components within the application to refer to when establishing a component state.

An authenticated connection to the Epic Sandbox server was established ( Supplementary Appendix 1 ) and the FHIR Resource requests for “Appointment,” “Patient,” and “MedicationRequest” were made to pull EHR data from the provided sandbox patient “Camila Lopez.”

Upon receiving the data from these requests, appointment data was filtered by date for the three closest future appointments and stored in the application state along with patient name, patient ID, and session authentication token. MedicationRequest returned a non-standardized medication name; therefore, an additional FHIR call (Medication) was made using the Epic medication reference ID. This FHIR call allowed for the application to retrieve the medication RxCUI, which was then used to lookup the RxNorm drug name and available dosages via the National Library of Medicine Clinical Table Search Service API for RxTerms. 26 27 28 After retrieving the RxNorm data, both MedicationRequest and RxTerms data were stored into the application state with the corresponding medication information.

To assist patients with medication names and dosages, the RxTerms API was leveraged to drive an autocomplete function. This API also allowed the application to capture the RxCUI of each medication. Changes to existing medications on the medication list, including notes about side effects, new dosages, or other notes, were also stored within an “edits” object associated with the respective medication that was changed. Medications that were removed from the list were flagged as “removed” within the “status” field.

To allow patients to print their home medication list, we used the “react-to-print” package, a utility that converts what is seen on a screen to a downloadable and printable .pdf file. Concurrently, a MedicationRequest FHIR package representing the patient-documented medication list, along with patient annotations to the medication list, is generated to be sent to a FHIR endpoint on a receiving system ( Supplementary Appendix 2 ).

### Functionality Guide

The final artifact was documentation of the expected functionality of a solution, to give collaborators an understanding of the conventions expressed via the wireframe. A functionality style guide ( Supplementary Appendix 3 ) was written that describes the function of each component of the application, the parent–child relationship between each component, and the flow of information from one component to the next.

## Results

**Fig. 1:**
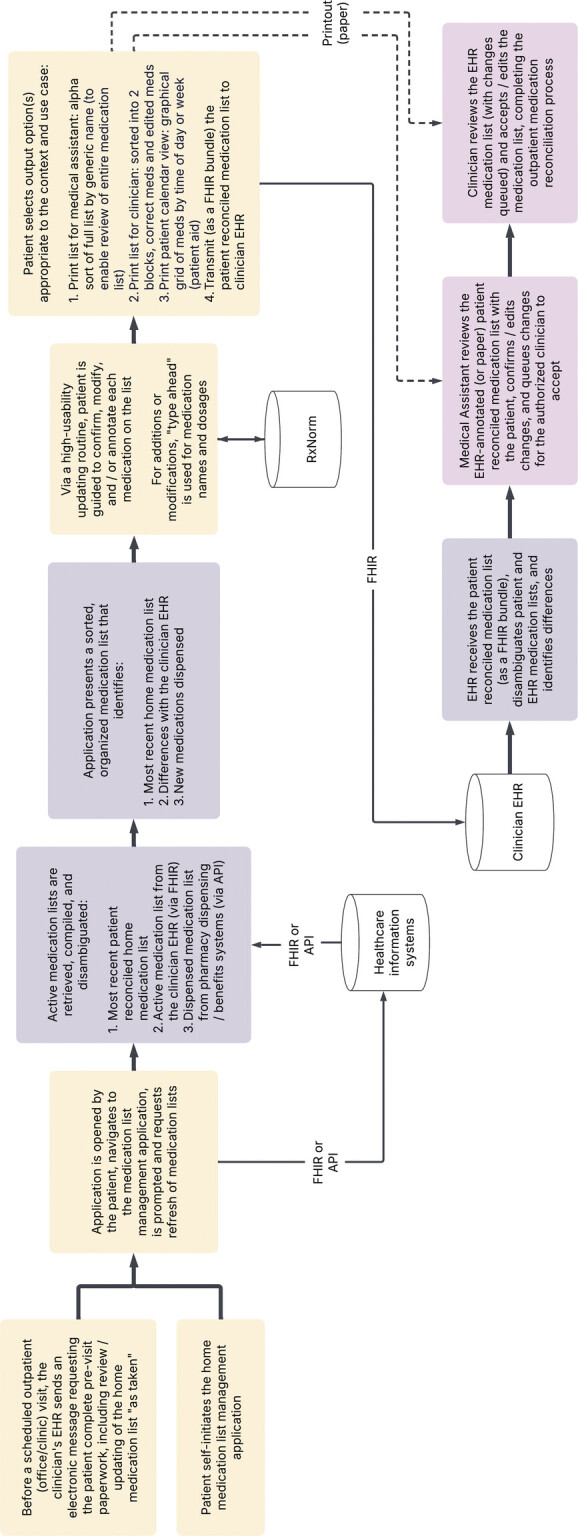
Conceptual Model. High-level model representing primary features of a patient-facing, patient-centric home medication list management system to facilitate office visit medication reconciliation. Blocks with a light tan background summarize patient-facing interactions. Gray blocks summarize functionality executed by information systems. Lavender blocks summarize interactions with clinicians. API, application programming interface; her, electronic health record; FHIR, Fast Healthcare Interoperability Resources; RxNorm, standardized terminology for clinical medications managed by the National Library of Medicine.

**Fig. 2:**
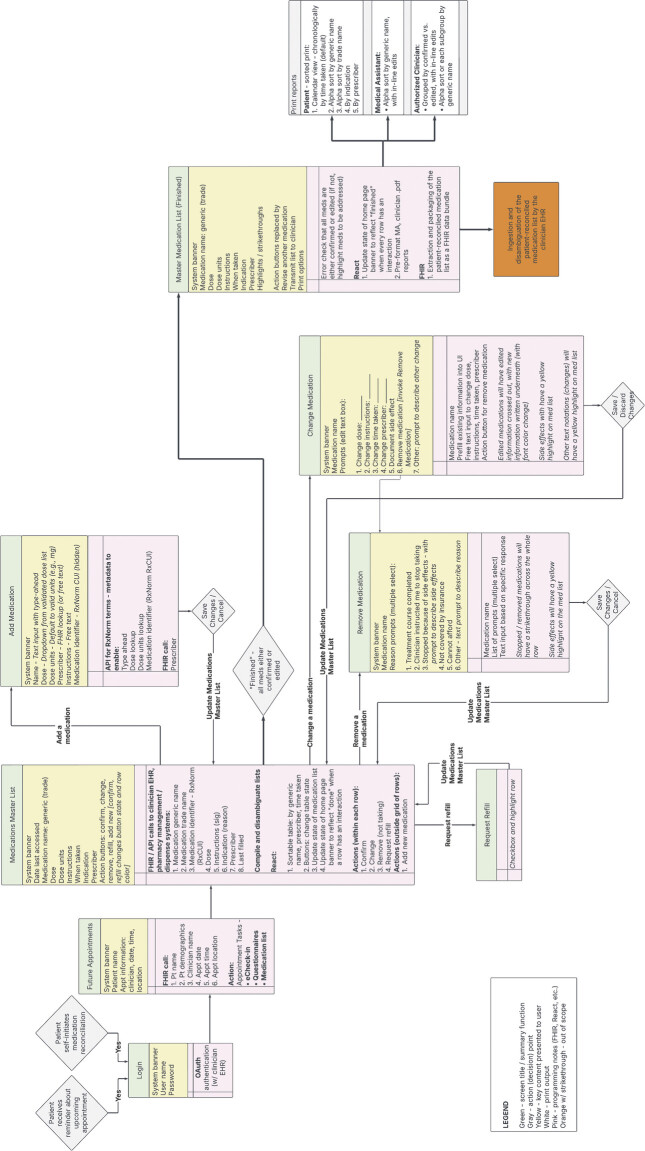
Data Architecture Model. This model includes representations of interactions among actors, patient user interface, expected workflows, key data, and functionality for a patient home medication list management application with medication data retrieval, patient-centric medication list management, and patient-reconciled medication list information forwarding functionality. API, application programming interface; FHIR, Fast Healthcare Interoperability Resources; RxNorm, standardized terminology for clinical medications managed by the National Library of Medicine.

**Fig. 3:**
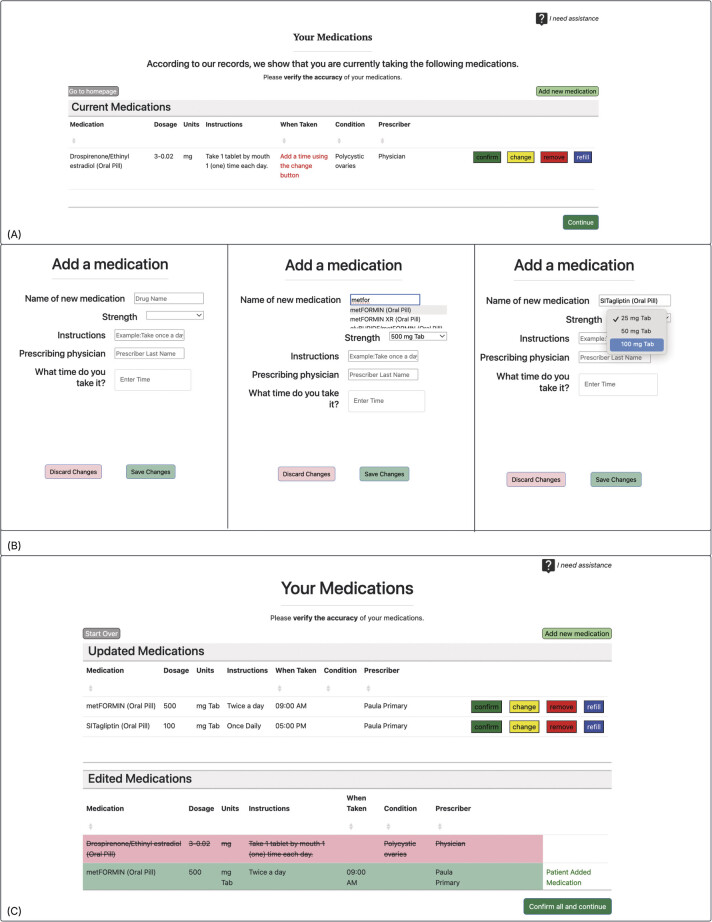
Wireframe screenshots. ( **A** ) The baseline, inaccurate medication list as provided through the Epic sandbox patient chart. ( **B** ) The screen that is displayed after clicking “Add a medication.” This form allows a patient to search for his or her medication, and prescribable dosages are automatically populated to the strength dropdown menu. ( **C** ) The updated medication list containing patient-generated edits.

Our modeling approach included the creation of four principal artifacts. These included a conceptual (high-level) model ( Fig. 1 ), a data architecture (detailed) model including representations of the interactions among actors, workflows, data, and functionality ( Fig. 2 ), a functionality (style) guide articulating the expectations and behaviors of the system ( Supplementary Appendix 3 ), and a high-fidelity end-to-end wireframe simulating a basic Personal Health Record (PHR) performing the functions defined in the model ( Fig. 3 ) ( *https://github.com/ndb77/EMET_Project/tree/main* ).

The conceptual model ( Fig. 1 ) has three primary human actors in the medication list management ecosystem: the patient (or surrogate) as the “source of truth” of the actual medications “as taken” by the patient, a medical assistant (the individual who rooms a patient at the time of an outpatient visit and conducts a preliminary review of the medication list), and the authorized clinician (the clinician who can legally commit changes that update a patient's medication list in the EHR). Regardless of whether access is provided via a patient portal to an EHR (i.e., a tethered PHR) or a standalone PHR application, basic patient-facing functionality includes the ability to annotate a medication list—either confirming the medication, adding a new medication to the list, changing the parameters (e.g., dose, dosing frequency) of a medication, or indicating that a medication is not being taken.

The data architecture model ( Fig. 2 ) identifies the data used to support the patient-facing functionality, including FHIR and other API queries for existing medication list data from EHR and pharmacy management systems, other interfaces with external systems, disambiguation of inconsistencies across medication lists, and export of a patient medication list reconciliation report for ingestion by an EHR system.

The end-to-end wireframe confirmed the ability to use available FHIR resources as modeled in the conceptual and data architecture models above. The proof-of-concept included functionality for patients to retrieve their medication list from an EHR system, document their home medications as taken, and export a FHIR data bundle representing the patient-reconciled home medication list, including annotations regarding discrepancies in prescription name, dosage, timing, and patient concerns about specific medications. The wireframe includes calls to the RxNorm system to provide the data needed to automatically format medications according to their RxTerms naming conventions so that medication data sent by the wireframe is consistent and inherently interoperable. 28 To reduce patient burden, the wireframe further leveraged RxNorm data to allow the patient to easily search for medication names (via type ahead) and medication-specific dosages. The output, a patient-annotated medication list, to be disambiguated and then reconciled at the time of an outpatient visit, contains standardized medication names and dosages along with patient annotations ready for ingestion by a corresponding EHR FHIR endpoint.

**Fig. 4:**
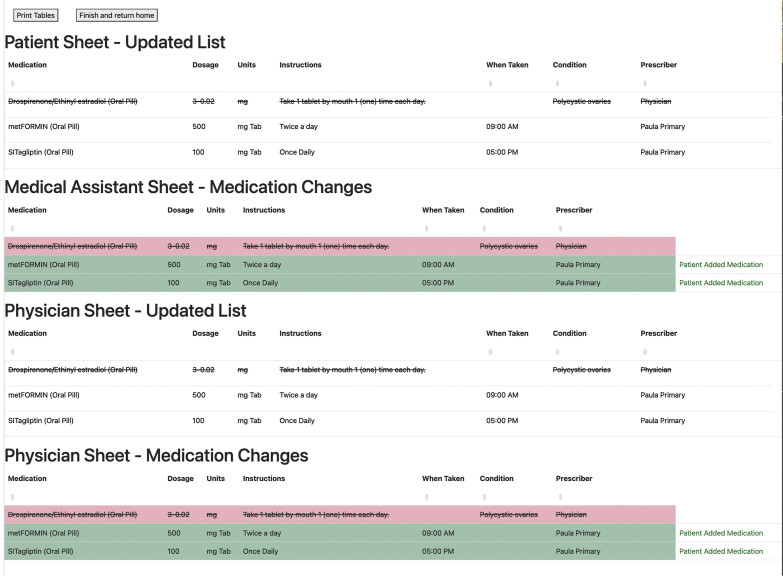
A printable .pdf medication list.

The wireframe was intentionally built to use FHIR API calls and to evaluate model readiness by connecting with the sandbox of a popular and mature EHR (Epic). During the development and testing process, we uncovered specific limitations that made it difficult to independently design applications to interface with existing medication reconciliation infrastructure. A major shortfall was the lack of synthetic data availability. In the Epic sandbox, there was only 1 patient with authentication credentials with a populated medication list (of only one medication). Second, while outpatient pharmacy dispense data are available via proprietary API services, companion FHIR endpoints to access pharmacy dispense data were not available. In addition, the functionality needed to ingest a patient-authored medication list that does not exist as a FHIR endpoint and paradigms to manage the disambiguation of multiple medication lists is similarly immature. Consequently, our wireframe includes the option to print three versions of the medication list containing different annotations pertinent to different members of a care team. The medical assistant view features alpha sorted medications by generic name, with visual highlighting of correct as listed vs. patient-annotated medications. The clinician print view grouped medications differently, with correct medications presented in one block and patient-annotated medications in a separate block ( Fig. 4 ).

## Discussion

Accurate maintenance of the patient medication list “as taken” is paramount for optimal patient care. 1 2 3 5 6 7 8 9 10 11 12 Although systematic, mature, and well-resourced processes exist to accomplish medication reconciliation associated with hospital transitions of care, quality medication reconciliation in the context of the routine, scheduled outpatient encounter is dependent on the individual with the least amount of time—the licensed clinician caring for the patient. As a prime opportunity to reduce clinician burden and improve patient care, the HL7 Electronic Health Record – Reducing Clinician Burden workgroup identified patient medication list management and outpatient medication reconciliation as a prime opportunity for the application of interoperability standards. Our project successfully modeled an ecosystem that supports a patient-centric approach to outpatient medication reconciliation and demonstrated a technical implementation leveraging FHIR-based interoperability through a high-fidelity wireframe.

A major limitation of the existing medication reconciliation ecosystem is a lack of consensus about how a medication reconciliation event should be digitally represented. This has led to the development of solutions that in part address specific slices of the medication reconciliation challenge but fail to be widely applicable across multiple systems. Bespoke solutions tend to be required not just for each EHR system but also for every individual healthcare entity. Compounding this is the inability to easily convey medication list data from one clinician to another, particularly among clinicians not aligned with a single healthcare entity and single EHR system. This results in duplicative effort, inconsistent and inaccurate medication lists, and a loss of understanding between patients and their clinicians regarding the medications that patients are taking.

Multiple groups have developed solutions to attempt to effectively generate an accurate patient medication list. MEDIvate is an example of a high usability iOS application and cloud architecture that supports the rapid transfer of an updated medication list, as per the provider EHR, to a patient's mobile device and provides resources for medication education upon discharge from the hospital. 29 This ensures that at the time of discharge, the patient medication list is aligned with the expectations of the prescribing provider. Similarly, Spratt et al developed a FHIR-based medication reconciliation application built into an EHR patient portal to connect patients with pharmacists and facilitate the medication reconciliation process. 29 30 Similarly, Ziminski et al created an application capable of aggregating information from multiple EHRs to generate a medication list free from duplications and reflective of what a health system believes are the patient's at-home medications. 31 Limitations common across these solutions are the dependency on the hospital-based EHR and availability of hospital resources (e.g., pharmacists) along with the inability of patients to directly input information about the medications they actually take.

Properly implemented, the patient-facing PHR provides the opportunity for patients to access, manage, and share their own healthcare information. PHR systems are well-positioned to facilitate patient-centric medication reconciliation as they facilitate direct patient input. Although the PHR tethered to a specific EHR can potentially leverage EHR functionality (e.g., direct access to the patient medication list as maintained in the EHR), patient management of the medication list for purposes of data sharing with clinicians who not using that EHR is severely constrained. Independent PHRs allow users to retain ownership of their healthcare data but struggle to be interoperable with EHRs. Previous work in this space includes the report by Saripalle et al who built a tethered prototype system for bidirectional data transferability between patient-maintained health data on a PHR and OpenEHR, an open source FHIR-compatible EHR. 32 The authors suggest that the benefits of interoperability outweigh the loss of personal data sovereignty. However, we propose that a properly implemented independent PHR framework can allow for both vendor agnostic interoperability for medication reconciliation and retention of health data ownership.

Presently, data interoperability for medication reconciliation through an independent PHR is difficult because there has been a lack of standardization on how to transmit and consume the pertinent data in a medication reconciliation event. HL7 consensus-based development of EHR and PHR Functional Profiles provides a pathway to specify content, dataflow, workflow, interoperability, and operational requirements as technical standards. A key purpose of our project has been to envision those requirements and bring them forward as an HL7 standards project. To this end, PSS-2452 has been accepted for development by the HL7 EHR Workgroup. 33 Based on the findings of our project, the intent is to extend the ISO/HL7 10781 Electronic Health Record System Functional Model R2.1 and the ISO/HL7 16527 Personal Health Record System Functional Model R2 to incorporate specifications supporting patient-centric medication list management and outpatient medication reconciliation. 21 22

Kashyap et al have called for a system that can generate an electronic Best Possible Medication History (eBPMH) through the aggregation of all EHR, pharmacy dispense system, pharmacy benefit manager, and patient-generated data. 34 Conformance to the Outpatient Medication Reconciliation Functional Profiles outlined in PSS-2452 will allow for either an EHR-tethered or -independent PHR to generate an eBPMH and participate in the medication reconciliation process, with the independent PHR also permitting patient ownership and independent use of their health data. Critically, by enabling patient participation in the electronic medication reconciliation process, we make the patient the “source of truth” concerning the medications the patient is actually taking at home. This potentially positions the patient, not the prescriber, as the supplier of the medication list to recipients (clinicians, pharmacies, pharmacy benefit managers, insurance companies, etc.) via a one-to-many broadcasting schema. With knowledge of an accurate home medication list, along with automatic identification and disambiguation of discrepancies, efficient and appropriate interventions can be taken to address at-home medication list errors.

The development of the proposed Functional Profiles will be a substantive step toward consensus among key stakeholders about how patient-facing medication list management and medication reconciliation events should be represented by PHRs, communicated via FHIR, and received by EHRs. Adoption of these Functional Profiles would enable the creation of an eBPMH. The reference model that we developed provides a structured framework with demonstration through a high-fidelity wireframe. The model can act as a guide for developers and establishes a foundation for further refinement. Of note, our investigation suggests the need for further development in the medication list management and reconciliation space. This includes the need for more robust synthetic data and EHR sandbox implementations for testing, a dedicated FHIR profile to consistently assemble a patient medication list across all EHR systems, FHIR endpoints for access to pharmacy dispense and pharmacy benefits data, and functionality to ingest, disambiguate, and present the patient-authored medication list for clinician reconciliation. Of note, the scope of the project was limited and did not include the encryption of data storage and other standard approaches to ensure data security and privacy in the modeling, elements that are needed in production-level solutions. We anticipate that developers will differentiate solutions based on usability and so included only minimal usability testing in the wireframe. Evaluating the practical utility of our model and impact on real-world medication reconciliation solutions will be an essential focus for future research.

## Conclusion

An accurate medication list “as taken” is paramount for optimal patient care. We developed a reference model for a FHIR-based patient home medication list management and outpatient clinical visit reconciliation application. Our modeling delineates dataflow, workflow, interoperability, and functionality requirements for an end-to-end, patient-centric, medication list management ecosystem. This modeling should help steer product development toward functionality that can increase usability and applicability of patient home medication list management while reducing the clinician burden of outpatient medication reconciliation.

## Clinical Relevance Statement

An accurate medication list “as taken” is paramount for optimal patient care. Medication reconciliation is a repetitive, burdensome, resource-intensive process that could be streamlined through digital solutions, improving list accuracy while reducing clinician burden. We developed a reference model for a FHIR-based patient home medication list management and outpatient clinical visit reconciliation application. Our modeling provides the basis for specification of extensions to the HL7 EHR and PHR functional models, delineating dataflow, workflow, interoperability, and functionality requirements for an end-to-end, patient-centric, medication list management ecosystem. This model may help to steer product development toward functionality that can increase usability and applicability of patient home medication list management while reducing the clinician burden of medication reconciliation at the time of an outpatient office visit.

## Multiple-Choice Questions

1. What was the purpose of this project?
  - To develop a fully functioning mobile health application.
  - To express the ideas and concepts of the Medication Reconciliation initiative of the HL7 Reducing Clinical Burden workgroup.
  - To identify stress points in the doctor–patient interaction when addressing medication adherence.
  - To advocate for the reduction of medications on a med list for elderly adults.
  **Correct Answer:** The correct answer is option c. The intention is for developers to be informed of the critical actions that need to be incorporated into a home medication list reconciliation application for the office (outpatient) visit context. At present, many applications allow for local storage of patient medication adherence (pill trackers); however, there does not exist a way for patients to send a patient-reconciled home medications list to their clinician's EHR. The ability to create, read, update, and delete items on a reconciled version of a patient medication list is currently communicable using FHIR resources. Despite communicability, the ability to push a patient-reconciled version of the patient's medication list “as taken” to a clinician's EHR remains be implemented.
2. What is the output of this medication reconciliation application?
  - A QR code containing a patient's home medication list.
  - An email containing the patient's home medication list.
  - A pdf printout and a JSON packet of a patient's home medication list.
  - Nothing, this application does not produce an output.
  **Correct Answer:** The correct answer is option c. This application outputs a pdf printout of a patient medication list and a FHIR-based JSON packet that describes actions taken during the patient reconciliation event. A JSON packet containing FHIR resources allows for a patient to agnostically submit a reconciled medication list to an accepting EHR, along with metadata containing actions taken on the medication list. The information within the pdf is intended to simply show a patient's active medication list, as described by the patient, to various members of a patient's care team. A pdf print out can allow patients to directly communicate their reconciled list to the care team and circumvent compatibility issues between the patients' application and the provider's EHR.

## References

[1] Coleman J J Pontefract S K Adverse drug reactions Clin Med (Lond) 2016 16 05 481 485 27697815 10.7861/clinmedicine.16-5-481 PMC6297296

[2] Naseralallah L Stewart D Price M Paudyal V Prevalence, contributing factors, and interventions to reduce medication errors in outpatient and ambulatory settings: a systematic review Int J Clin Pharm 2023 45 06 1359 1377 37682400 10.1007/s11096-023-01626-5 PMC10682158

[3] Tariq R A Vashisht R Sinha A Medication Dispensing Errors and PreventionStatPearls [Internet].StatPearls Publishing 2024 30085607

[4] By the 2023 American Geriatrics Society Beers Criteria® Update Expert Panel American Geriatrics Society 2023 updated AGS Beers Criteria® for potentially inappropriate medication use in older adults J Am Geriatr Soc 2023 71 07 2052 2081 37139824 10.1111/jgs.18372 PMC12478568

[5] Barnsteiner J H Medication Reconciliation. Patient Safety and Quality: An Evidence-Based Handbook for Nurses Agency for Healthcare Research and Quality (US) 2008 21328752

[6] Young R A Fulda K G Espinoza A Ambulatory medication safety in primary care: a systematic review J Am Board Fam Med 2022 35 03 610 628 35641040 10.3122/jabfm.2022.03.210334 PMC9730343

[7] Caglar S Henneman P L Blank F S Smithline H A Henneman E A Emergency department medication lists are not accurate J Emerg Med 2011 40 06 613 616 18829201 10.1016/j.jemermed.2008.02.060

[8] Barnsteiner J H Medication reconciliation: transfer of medication information across settings-keeping it free from error Am J Nurs 2005 105(3, suppl):31 36, quiz 48–51 10.1097/00000446-200503001-00007 15802996

[9] Laatikainen O Sneck S Turpeinen M The risks and outcomes resulting from medication errors reported in the Finnish tertiary care units: a cross-sectional retrospective register study Front Pharmacol 2020 10 1571 32009966 10.3389/fphar.2019.01571 PMC6978730

[10] Moore C Wisnivesky J Williams S McGinn T Medical errors related to discontinuity of care from an inpatient to an outpatient setting J Gen Intern Med 2003 18 08 646 651 12911647 10.1046/j.1525-1497.2003.20722.x PMC1494907

[11] Säfholm S Bondesson Å Modig S Medication errors in primary health care records; a cross-sectional study in Southern Sweden BMC Fam Pract 2019 20 01 110 31362701 10.1186/s12875-019-1001-0 PMC6668157

[12] Neiman A B Ruppar T Ho M CDC grand rounds: improving medication adherence for chronic disease management—innovations and opportunities MMWR Morb Mortal Wkly Rep 2017 66 45 1248 1251 29145353 10.15585/mmwr.mm6645a2 PMC5726246

[13] Baughman A W Triantafylidis L K O'Neil N Improving medication reconciliation with comprehensive evaluation at a veterans affairs skilled nursing facility Jt Comm J Qual Patient Saf 2021 47 10 646 653 34244044 10.1016/j.jcjq.2021.06.001

[14] Meguerditchian A N Krotneva S Reidel K Huang A Tamblyn R Medication reconciliation at admission and discharge: a time and motion study BMC Health Serv Res 2013 13 485 24261516 10.1186/1472-6963-13-485 PMC3842651

[15] Overview-arch - FHIR v6.0.0-ballot2. Accessed February 9, 2025 at:https://build.fhir.org/overview-arch.html

[16] HL7 Balloting. 2025. Accessed at:https://confluence.hl7.org/display/HL7/HL7+Balloting

[17] 21 ^st^ Century Cures Act. 2016. Accessed at: https://www.gpo.gov/fdsys/pkg/BILLS-114hr34enr/pdf/BILLS-114hr34enr.pdf

[18] HL7 As Is To Be Document. 2022. Accessed at:https://confluence.hl7.org/download/attachments/104568480/Scenario%20v31%20As%20Is_To%20Be%20March%202022%20DRAFT%20Final.docx?version=3&modificationDate=1647198333064&api=v2

[19] Reducing Clinician Burden. Confluence Page. 2025. Accessed at:https://confluence.hl7.org/spaces/EHR/pages/104568480/Reducing+Clinician+Burden+RCB

[20] HL7 Reconciled Medication List Presentation. 2021. Accessed at:https://confluence.hl7.org/spaces/EHR/pages/104568480/Reducing+Clinician+Burden+RCB?preview=%2F104568480%2F116461224%2FSchlossman+RML+Presentation_14JUN2021_v3.pdf

[21] Health informatics—HL7 Electronic Health Record-System Functional Model, Release 2.1 (EHR FM). 2019. Accessed February 19, 2025 at:https://www.iso.org/obp/ui/en/#iso:std:iso:10781:ed-1:v1:en

[22] Health informatics—HL7 Personal Health Record System Functional Model, Release 2 (PHR-S FM). 2019. Accessed February 19, 2025 at:https://www.iso.org/obp/ui/en/#iso:std:iso:16527:ed-1:v1:en

[23] Medication List Management and Reconciliation. 2023. Accessed at:https://confluence.hl7.org/spaces/EHR/pages/175612088/2023-07-12+Medication+List+Management+and+Reconciliation+Meetings

[24] Standardized Medication Profile. 2021. Accessed February 19, 2025 at:https://www.ncpdp.org/NCPDP/media/pdf/WhitePaper/Standardization-Medication-Profile-White-Paper.pdf?ext=.pdf

[25] United States Core Data for Interoperability (USCDI). 2022. Accessed February 9, 2025 at:https://www.healthit.gov/isp/united-states-core-data-interoperability-uscdi#uscdi-v3

[26] Home - Epic on FHIR. 2025. Accessed February 9, 2025 at:https://fhir.epic.com/

[27] RxNorm Overview. 2005. Accessed February 9, 2025 at:https://www.nlm.nih.gov/research/umls/rxnorm/overview.html

[28] RxTerms API - APIs. Accessed February 9, 2025 at:https://lhncbc.nlm.nih.gov/RxNav/APIs/RxTermsAPIs.html#:~:text=The%20RxTerms%20API%20is%20a,to%20use%20the%20RxTerms%20API

[29] Coons J C Patel R Coley K C Design and testing of MEDIvate, a mobile app to achieve medication list portability using HL-7 FHIR Journal of the American Pharmacists Association: JAPhA 2019 59 S78 30737102 10.1016/j.japh.2019.01.001 PMC6411446

[30] Spratt S E Ravneberg D Derstine B Granger B B Feasibility of electronic health record integration of a SMART application to facilitate patient-provider communication for medication management Comput Inform Nurs 2022 40 08 538 546 35234708 10.1097/CIN.0000000000000891

[31] Ziminski T Demurjian S Agresta T Extending the fast healthcare interoperability resources (FHIR) with meta resourcesProceedings of the 16th International Conference on Software Technologies. SCITEPRESS - Science and Technology Publications 2021

[32] Saripalle R Runyan C Russell M Using HL7 FHIR to achieve interoperability in patient health record J Biomed Inform 2019 94 103188 31063828 10.1016/j.jbi.2019.103188

[33] Outpatient Medication Reconciliation. 2024. Accessed at:https://jira.hl7.org/projects/PSS/issues/PSS-2452?filter=allissues

[34] Kashyap N Jeffery S Agresta T From MedWreck to MedRec: a call to action to improve medication reconciliation Appl Clin Inform 2024 15 02 230 233 37748724 10.1055/a-2181-1847 PMC10972679

