# Supplementary material for: Framework for Improving Patient Safety: Reference Model for FHIR-Enabled, Patient-Centric Home Medication List Management and Medication Reconciliation
Source: Appl Clin Inform. 2025 Sep 19;16(4):1136–45. doi: 10.1055/a-2599-4135 (PMC12449101; doi:10.1055/a-2599-4135)
Supplement: Supplementary file 1 — Supplementary Material [file 10-1055-a-2599-4135_27058333.pdf]

Supplementary Appendix 1

Before the patient medication list and other data could be viewed, an authenticated connection to the Epic Sandbox server needed to be established. To do this, an account was created on the "Epic on FHIR" website, including FHIR app registration using their registration portal. Through this portal, a "non-production client ID" was obtained, and the following FHIR(R4) API calls were invoked:

1. PATIENT.READ
2. PATIENT.SEARCH
3. APPOINTMENT.READ
4. APPOINTMENT.SEARCH
5. MEDICATIONREQUEST.READ
6. MEDICATIONREQUEST.SEARCH
7. MEDICATION.READ

Additionally, the redirect URI was set to "localhost:3000/home," as this was the intended redirect page after passing through the Epic authentication page. Lastly, the test patient credentials were obtained through the Epic Sandbox test data page. This project used the test user credentials for "Camila Lopez."

After using the Epic test user credentials, FHIR requests were executed for the following resources:

1. Appointment[1]
2. Patient
3. MedicationRequest (active medications only)

Supplementary Appendix 2

JSON is a human and machine-readable text formatting widely used to transmit data, including FHIR data. By creating a JSON output, it allows for end users to have freedom to choose where to send or store their data. An example of the .json output generated by the application can be found at https://github.com/ndb77/EMET_Project/blob/main/example-output.json.

Supplementary Appendix 3: Style Guide

Patient Medication Reconciliation
Solution Functionality (Style) Guide

Application Folder Structure

The code for this project is stored in a Github repository and is available for public use and replication. The project folder, "medication-reconciliation," contains the following folder structure, which was automatically generated using the "create-react-app" package [26]:

1. node_modules
2. public
3. src
   a. bootstrap
   b. components
   c. css
   d. hooks
   e. img
   f. App.js
   g. Index.js
   h. Store.js
4. package-lock.json
5. package.json
6. README.md
7. tsconfig.json

Folder and File Descriptions

node_modules: contains the code for the various installed packages and their dependencies.

public: contains static files that are unchanged, boilerplate files that were automatically generated from the "create-react-app" package.

package.json: a file that specifies all the dependencies used in this project, their version numbers, as well as overrides that allow for the use of Typescript components, scripts to run, and usable browsers.

tsconfig.json: a file used to enable the usage of Typescript components in this project.

src (source files):

bootstrap: contains files that were automatically generated using Bootstrap Studio, including minified css and javascript code used by the application.

components: contains all the logic for the different screens within the application and the various components that make up each screen. Each component contains HTML code that was initially created through Bootstrap Studio, and subsequently individually modified as needed.

css: contains the "styles.css" file which contains embedded styling conventions used throughout the application.

hooks: contains re-usable functions that were used for things such as fetching API data, autocomplete functionality, and form handling.

img: contains all the images used in the application.

App.js: defines the accessible pages of the application as well as the Route paths used to reach those pages. Routing is made possible through the "react-router-dom" package.

Index.js: the very top level of the file hierarchy and the entry point file of the application. "Index.js" renders "App.js" and makes the information defined in "Store.js" and the Routes defined in "App.js" accessible throughout the entire application.

Store.js: contains logic pertaining to the locally available state information that is accessible from all parts of the

[1] Epic Sandbox did not list future appointments for this test patient; therefore, for this project we used the Appointment resource for a different patient. For the patient ID and Bearer token providing the future appointment data, the Appointment. Search API function of the Epic on FHIR website was invoked via the "Try It" function. The patient ID is in the "patient" box, and the Bearer token is provided after clicking the "Raw Request" dropdown.

 © 2025. The Author(s).

application. This file contains information like the initial data structure of the medication list, appointment list, and user information as well as functions like "saveMedication," "setUser," or "editMedication" that can mutate these data.

Components

LoginPage.js: The first page of the application found at the route "/".

- Contains "FhirSignInButton.js" and uses the "fhirclient" package.
- "FhirSignInButton.js" causes the user to be automatically redirected to the Epic authentication page and attempts to create an authorization link using the provided ClientID, taken from the Epic FHIR app registration portal, a redirectUri, which points to the next intended screen in the application, in this case this is set to "http://localhost:3000/home," and the FHIR server url, which is https://fhir.epic.com/interconnect-fhir-oauth/api/FHIR/R4 for this case.
- At the Epic authentication page, the Epic sandbox credentials used are Username: fhircamila and Password: epicepic1, followed by redirect to our application's home page.

HomePage.js: The second page of the application, found at the route "/home," completes three major operations upon page render.

- Creates FHIR requests for Patient, MedicationRequest, and Appointment resources.
- Creates a FHIR call for Medication resources for all medications within the MedicationRequest resource.
- Uses the NLM API to retrieve RxNorm name and dosage information for each medication.

Using a variety of functions, an object is created containing the following structure:

1. listID—an id value specific to the applications medication list. This number iterates according to the medication list length + 1.
2. confirmStatus—whether or not this medication is edited, unedited, or confirmed as accurate.
3. medicationReferenceDisplay—the medication name.
4. medicationWhenTaken—when the medication is taken. This value is set by the user and is initially set as an empty string.
5. medicationReferenceId—the reference ID value as given by Epic.
6. dosageInstruction—an object containing the dosage information, such as instructions, dosage, rate.
7. reasonCode—an object containing the diagnosis code, as given by Epic, which can contain information such as the ICD-10 code.
8. Requester—the display name of the prescriber.
9. Status—whether the medication is active, edited, or removed from the medication list.
10. validityPeriodStart—when the medication was started.
11. resource—an object containing the complete MedicationRequest resource as given by Epic.
12. rxNormData—an object containing the drug name, dosage, dosage units, and the full RxNorm query response.
13. edits—an object containing edits to the medication list such as new instructions, new dosages, side effects, and other notes.

Each medication is stored into local storage in a medication list object, the closest three appointments are stored in an appointments list object, and the user information is stored in a user object. Additionally, this component renders the available upcoming appointments within a scrollable list.

CurrentMedicationList.js: Renders link buttons to "Add a new medication," "Go to homepage," or "Continue" to the next page. Additionally, this component renders the "MedicationListTableComponent."

MedicationListTableComponent, MedicationListTableHeadComponent, MedicationListTable, MedicationListTableBodyButton: These components work together to allow for medications to be rendered into a table and to be organized by alpha-numeric order according to the heading category that is clicked.

- "MedicationListTableComponent" sets the column labels, provides a "handleSort" function, collects the medication data that was previously stored into local storage, and provides a "medicationListVersion," which decides if the rendered list is the current list, the updated list, or the edited list.
- "MedicationListTableHeadComponent" receives the column labels, the "handleSort" function, and the "medicationListVersion" while "MedicationListTable" receives the column data, table data, and the "medicationListVersion."
- "MedicationListTable" populates each table row with information taken from the stored data elements described above and renders a "MedicationListTableBodyButton" component which is an interactive button to indicate a medication as "Confirm," "Change," "Remove," and "Refill." Clicking these buttons will cause the selected medication's "status" to change as handled by the logic within "MedicationListTableBodyButton."

ChangeMedicationForm.js: Clicking the "Change" button for a medication will take the user to a page rendered by this component. This component contains two child components: "ChangeMedicationFormReason.js" and "ChangeMedicationFormReason2.js."

- "ChangeMedicationFormReason.js" takes a user selection, such as "I need to change something," and passes the selection to its parent, which then is sent to "ChangeMedicationFormReason2.js."
- "ChangeMedicationFormReason2.js" renders the second form page according to the selection from the first page. After the second form page is filled out and submitted, the changes are saved into the selected medication's "edits" object and the user is sent to the updated medications list page.

AddMedicationPage.js: Clicking the "Add a medication" button will take the user to a page rendered by this component

 © 2025. The Author(s).

which contains one child component, “AddMedicationMenu.js.” The children of “AddMedicationMenu.js” include “AddMedicationMenu.js,” “AddMedicationSearchBar.js,” “AddMedicationStrengthList.js,” “AddMedicationInstructions.js,” “AddMedicationPrescriber.js,” and “AddMedicationWhenTaken.js.” The search bar takes the user keystrokes and sends this information to the parent component that uses the NLM RxTerms API to provide data to autocomplete medication names and prescribable doses, and organizes the results into a list with format: [[medication name], [prescribable doses], [RxCUIS]]. The result list is sent back to the search bar and the medication names are rendered to the dropdown. Upon medication name selection, the corresponding prescribable doses are sent to the strength list dropdown to be rendered. Medication name and dosage selection are text-cleaned using various helper functions to store to individual variables within the parent component. The instructions input and prescriber input are simple text inputs that take the users keystrokes and send them to the parent component to be stored. “AddMedicationWhenTaken.js” uses the react package “@mui/x-date-pickers” and takes a 12-hour time selection from the user and sends it to the parent to be stored as a variable. Clicking the “Save Changes” button will create a new medication object on the medication list within the local storage and send the user to the updated medication list page.

UpdatedMedicationList.js: renders the updated medication list by passing the property of “updated” to its child component “MedicationListTable.js.” This causes the component to render an updated and edited medication list that renders the updated medication list in the upper table, and the edited medication list with appropriate styling such as cross-outs and labels, in the lower table. Clicking the “Confirm all and continue” button takes the user to the printout page.

PrintMedicationsPage.js: composed of several “PrintPageTable.js” children that are intended for the patient and the different actors on the patient’s care team.

- Because “react-to-print” requires components to be represented as forwardRef, each “PrintPageTable.js” is composed of a “MedicationListTableHeadComponent” and “MedicationListTable” that are exported to the parent component as a forwardRef.
- Clicking the “Print Tables” button prompts the user to save the tables as a printable PDF file.
- Clicking “Finish and return home” redirects the user to the home page and a data object is printed out within the console containing the medication list along with a FHIR MedicationRequest resource that can be sent to FHIR-data accepting systems.

 © 2025. The Author(s).
